# The physical and psychiatric health conditions related to autism genetic scores, across genetic ancestries, sexes and age-groups in electronic health records

**DOI:** 10.1186/s11689-023-09485-x

**Published:** 2023-06-16

**Authors:** Maria Niarchou, Tyne Miller-Fleming, Beth A. Malow, Lea K. Davis

**Affiliations:** 1 Vanderbilt Genetics Institute, Vanderbilt University Medical Center, Nashville, TN USA; 2 Division of Genetic Medicine, Vanderbilt University Medical Center, Nashville, TN USA; 3 Sleep Disorders Division, Department of Neurology, Vanderbilt University Medical Center, Nashville, TN USA; 4 Division of Neurology, Pharmacology and Special Education, Vanderbilt Kennedy Center, Vanderbilt University Medical Center, Nashville, TN USA; 5 Department of Biomedical Informatics, Vanderbilt University Medical Center, Nashville, TN USA; 6 Department of Psychiatry and Behavioral Sciences, Vanderbilt University Medical Center, Nashville, TN USA; 7 Department of Molecular Physiology and Biophysics, Vanderbilt University, Nashville, TN USA

**Keywords:** Autism, Polygenic score, PheWAS, Electronic Health Records

## Abstract

**Background:**

Although polygenic scores (PGS) for autism have been related to various psychiatric and medical conditions, most studies to date have been conducted in research ascertained populations. We aimed to identify the psychiatric and physical conditions associated with autism PGS in a health care setting.

**Methods:**

We computed PGS for 12,383 unrelated participants of African genetic ancestry (AF) and 65,363 unrelated participants of European genetic ancestry (EU) from Vanderbilt’s de-identified biobank. Next, we performed phenome wide association studies of the autism PGS within these two genetic ancestries.

**Results:**

Seven associations surpassed the Bonferroni adjusted threshold for statistical significance (*p* = 0.05/1374 = 3.6 × 10^−5^) in the EU participants, including mood disorders (OR (95%CI) = 1.08(1.05 to 1.10), *p* = 1.0 × 10^−10^), autism (OR (95%CI) = 1.34(1.24 to 1.43), *p* = 1.2 × 10^–9^), and breast cancer (OR (95%CI) = 1.09(1.05 to 1.14), 2.6 × 10^−5^). There was no statistical evidence for PGS-phenotype associations in the AF participants. Conditioning on the diagnosis of autism or on median body mass index (BMI) did not impact the strength of the reported associations. Although we observed some sex differences in the pattern of associations, there was no significant interaction between sex and autism PGS. Finally, the associations between autism PGS and autism diagnosis were stronger in childhood and adolescence, while the associations with mood disorders and breast cancer were stronger in adulthood.

**Discussion:**

Our findings indicate that autism PGS is not only related to autism diagnosis but may also be related to adult-onset conditions, including mood disorders and some cancers.

**Conclusions:**

Our study raises the hypothesis that genes associated with autism may also increase the risk for cancers later in life. Future studies are necessary to replicate and extend our findings.

**Supplementary Information:**

The online version contains supplementary material available at 10.1186/s11689-023-09485-x.

## Background

Autism is a heterogeneous and complex neurodevelopmental diagnosis that is characterized by difficulty with certain social interactions, restricted interests and/or repetitive behaviors, as well as features that affect school and work performance and may impact other areas of life. While language delay or difficulty is often a feature of autism, there is marked variation, in language and cognitive development, as well as variation in co-occurring psychiatric and medical conditions [26].

Co-occurring conditions are estimated to affect at least 70% of people with autism [20]. A recent meta-analysis provided pooled prevalence estimates of 28% for attention deficit hyperactivity disorder, 20% for anxiety disorders, 13% for sleep related disorders, 11% for depressive disorders, 9% for obsessive–compulsive disorder and 5% for bipolar disorders and 4% for schizophrenia spectrum disorders [20]. Other commonly reported medical conditions include infections, obesity, neurologic, and immunologic conditions [8, 10]. Recent reports suggest that long diagnostic delays may be accompanied by “masking” or “camouflaging”, which can have detrimental effects on lifetime mental health [1, 2]. Importantly, genetic signatures of autism can be leveraged to disentangle the relationship between biologically mediated co-occurring conditions and those that co-occur as a consequence of the common experiences of autistic people.

Genome-Wide Association studies (GWAS) demonstrate that autism is polygenic, i.e., it is associated with thousands of genetic variants of small effect [13]. The effect of these polymorphisms can be summed to result in a cumulative polygenic score for autism. Polygenic scores do not map perfectly to autism diagnoses. Autism as a clinical condition results from a complex interplay of genetic and non-genetic factors and receiving a diagnosis of autism also requires access to appropriate expertise. PGS for autism capture only part of the common variant genetic contribution to autism [15]. Some studies have shown that autism is not only phenotypically, but also genetically correlated with other neurodevelopmental conditions, including ADHD, major depression, and positively correlated with intelligence and educational attainment [3, 5, 13]. Thus, PGS are a useful tool to investigate whether and how genes associated with autism are also related to other psychiatric and medical conditions.

The majority of studies using PGS, however, have only tested the associations between autism PGS and co-occurring conditions in volunteer or research ascertained settings, rather than in health care settings. For example, a study in 334,976 volunteer participants from the UK biobank tested the associations between autism PGS and a number of general health and mental health characteristics and found associations with eight general health related outcomes and one mental health related outcome [22]. However, this study did not examine a wide variety of clinical diagnoses, nor did it examine how associations may change in different age groups, or across genetic ancestries.

The wealth of clinical information in Electronic Health Records (EHR) linked with genetic data provides the ability to conduct phenome-wide association scans (pheWAS) across a wide range of psychiatric and medical conditions. Using Vanderbilt’s biobank (BioVU) that is linked to de-identified medical records, we conducted a pheWAS to identify the physical and mental health conditions that are associated with autism PGS across genetic ancestries, sexes, and age-groups.

## Methods

### Vanderbilt University Medical Center EHR

The project was approved by Vanderbilt’s Institutional Review Board (IRB) (#190418). The phenotypic data originated from the synthetic derivative (SD), a de-identified clinical data repository of the EHR at VUMC, that includes records of over 3.2 million patients, and is updated regularly. The data includes billing codes from the International Classification of Diseases, 9th and 10th editions (ICD-9 and ICD-10), laboratory values, medication history, Current Procedural Terminology (CPT) codes, and clinical reports. BioVU is the associated biobank that accrues DNA samples from more than 280,000 of these patients [28].

### Genotyping data

The genotype data for a total of 94,474 samples were imputed using the Michigan Imputation server [9] and the Haplotype Reference Consortium (HRC) panel. The data were filtered to biallelic Single Nucleotide Polymorphisms (SNPs) only, and filtered to include SNPs with an *R*^2^ > = 0.3, and minor allele frequency > = 0.005. SNPs with a Hardy–Weinberg Equilibrium *P* value < 10e^−10^ were removed. To assign primarily European genetic ancestries as well as African ancestries, principal components analysis (PCA) was performed including all samples and the 1000 genomes reference populations. Details on ancestry assignment, imputation, and further quality control procedures are described elsewhere [11]. Cryptic relatedness was addressed by removing one individual of each pair for which pihat > 0.2. The final samples included 12,383 unrelated participants (median age of record (mean(SD) = 39.2(21.2), 61%females) of African (AF) ancestries and 65,363 unrelated participants of European (EU) ancestries (median age of record (mean(SD) = 48.4(22.2), 55.6% females) (also see Table 1).

**Table 1:** Basic demographics of the samples

	European genetic ancestries	African genetic ancestries
Sample size	65,363	12,383
% Females	55.6	61%
Median age of EHR record	Mean = 48.4, SD = 22.2	Mean = 38.5, SD = 21.1
Number of ICD codes	Mean = 260.22, SD = 379.85	Mean = 249.33, SD = 414.3
Length of record	Mean = 3651.4, SD = 2586.3	Mean = 3618.0, SD = 2647.2

*Abbreviations*: *EHR* electronic health records, *ICD* International Classification of Diseases record, *length of record* days from first to last date (ICD) code in the EHR, *number of ICD codes* the total number of ICD codes per individual

### Autism polygenic score

We constructed PGS based on the latest publicly available autism GWAS summary statistics [13]. The autism GWAS (training set) included 18,381 cases and 27,929 controls. The PGS per BioVU participant was computed using a continuous shrinkage prior (CS) to SNP effect sizes using the PRS_CS software [12] for participants of EU ancestries and the PRS_CSx [29] for participants of AF ancestries.

### Statistical analyses

The PGS were *Z*-score standardized with a mean of 0 and a standard deviation of 1. We ran two ancestry stratified PheWAS, one in the participants of EU genetic ancestries and and one in the participants of AF genetic ancestries. Outcomes in the PheWAS are based on phecodes that are comprised of ICD-codes grouped based on similarity [6]. The PheWAS analyses included 648 phecodes for the AF ancestry participants and 1374 phecodes for the EU ancestry participants with a minimum of 100 case patients. Individuals are assigned as a “case” for each phecode outcome if there are at least two instances of a corresponding ICD code map v1.2 [30] within their health record. Controls are assigned by the absence of the case defining ICD codes. Fewer phecodes met the minimum case number for inclusion in the AF ancestry population due to the smaller total number of individuals. PheWAS covariates included sex (defined as sex reported in the EHR), median age for an individual’s the medical record, current age (to control for cohort effects), and top ten principal components generated from genotype data (to control for population stratification). The *p* value threshold for statistical significance was Bonferroni corrected for multiple testing based on the number of tests in each PheWAS (i.e., 0.05/648 tests in the AF genetic ancestry = 7.7 × 10^−5^, and 0.05/1374 tests in the EU ancestry = 3.6 × 10^−5^). There were no statistically significant associations in the AF genetic ancestry, and therefore, no further analyses were conducted. Two conditional analyses were performed in which we further co-varied for (1) median BMI and (2) autism diagnosis in the EU ancestry.

We also conducted a sex stratified PheWAS in the EU ancestry sample. We then tested for significant differences in effect estimates between males and females for the phenotypes with evidence of a main effect in either sex, using an interaction term applied to the PheWAS model (Sex * PGS).

As an exploratory analysis, we tested associations of the autism PGS across different age groups. The age bin selection was based approximate life stages described in a previous study [25]. Specifically, the selected age (in years) bins were: 0 to 11 (children), 12 to 18 (teens), 19 to 25 (young adults, and college-age students), 26 to 40 (adults), 41 to 60 (middle aged adults), and 61 to 100 (older adults). Each PheWAS was adjusted for sex, median EHR age, current age and 10 PCs.

## Results

### African genetic ancestry population

#### PheWAS

**Fig. 1:**
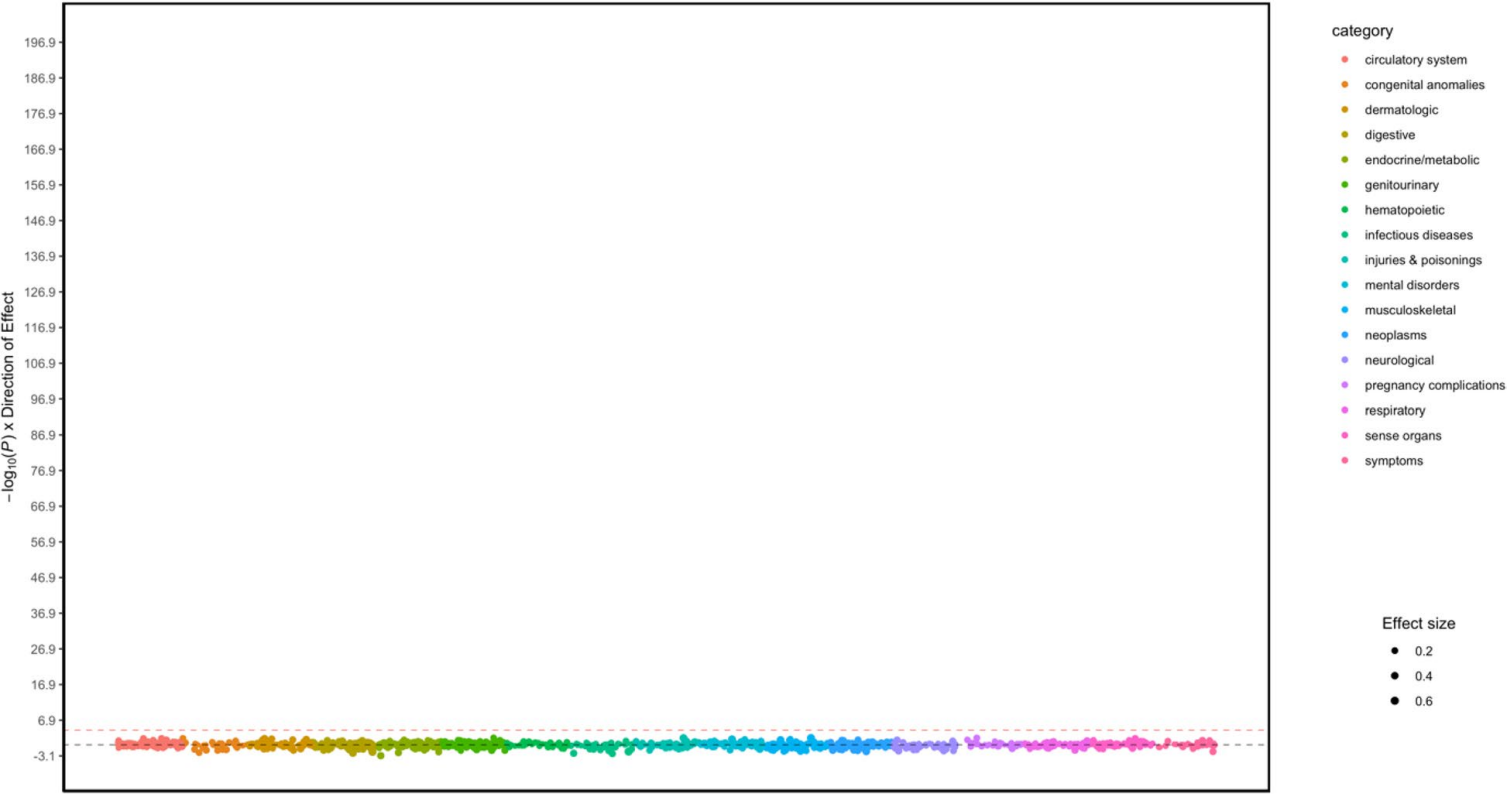
Manhattan plot of pheWAS results for the autism PGS in individuals of African genetic ancestries. The *y*-axis shows the -log10 transformed *p* values multiplied by the direction of effect. The dots represent the phecodes which are grouped along the *X* axis by phecode category. Size of the dot corresponds to the effect size. The colors of the dots indicate the phecode categories, which are explained in the figure. The dotted red line signifies the Bonferroni-corrected threshold for statistical significance

No associations passed the Bonferroni significance threshold (*p* = 0.05/648 tests = 7.7 × 10^−5^). Due to the lack of significant associations, no further analyses were performed (Table 2, Fig. 1, Supplementary Table S1).

**Table 2:** Summary of PheWAS results

Population	Bonferroni significant associations? (yes/no)	Phenotype	OR	95%CI	*p* value
AF	No				
EU	Yes	Mood disorders	1.08	1.05 to 1.10	1.0 × 10^−10^
		Autism	1.34	1.24 to 1.43	1.2 × 10^−9^
		Depression	1.07	1.04 to 1.09	3.1 × 10^−8^
		Pervasive developmental disorders	1.15	1.10 to 1.20	2.7 × 10^−7^
		Malignant neoplasm of female breast	1.10	1.05 to 1.14	2.5 × 10^−5^
		Breast cancer [female]	1.09	1.05 to 1.14	2.6 × 10^−5^
		Breast cancer	1.09	1.05 to 1.24	2.7 × 10^−5^

*Abbreviations*: *AF* African genetic ancestries, *EU* European genetic ancestries, *OR* odds ratio, *95%CI* 95% confidence intervals

### European genetic ancestry population

#### PheWAS

**Fig. 2:**
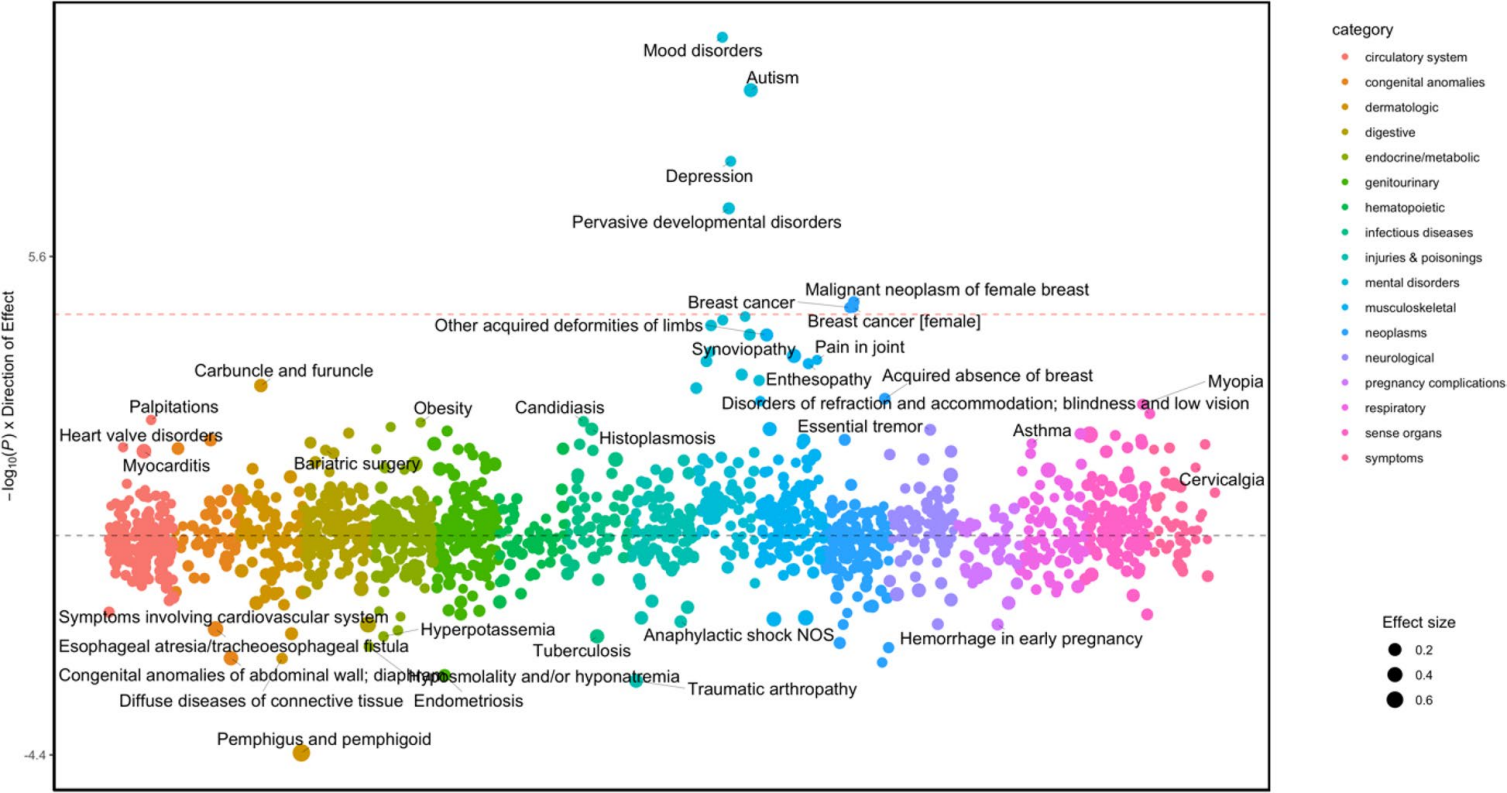
Manhattan plot of autism PGS in individuals of European genetic ancestries. The y-axis shows the -log10 transformed *p* values multiplied by the direction of effect. The dots represent the phecodes which are grouped along the *X* axis by phecode category. Size of the dot corresponds to the effect size. The colors of the dots indicate the phecode categories, which are explained in the figure. The dotted red line signifies the Bonferroni-corrected threshold for statistical significance

Seven associations surpassed the Bonferroni significance threshold (*p* = 0.05/1374 = 3.6 × 10^−5^), including mood disorders (OR (95%CI) = 1.08(1.05 to 1.10), *p* = 1.0 × 10^−10^), autism (OR (95%CI) = 1.34(1.24 to 1.43), *p* = 1.2 × 10^−9^), breast cancer (OR (95%CI) = 1.09(1.05 to 1.14), 2.6 × 10^−5^), depression (OR (95%CI) = 1.07(1.04 to 1.09), *p* = 3.1 × 10^−8^, pervasive developmental disorders (OR (95%CI) = 1.15(1.10 to 1.20), *p* = 2.7 × 10^−7^), malignant neoplasm of female breast (OR (95%CI) = 1.10(1.05 to 1.14), *p* = 2.1 × 10^−5^), and breast cancer [female] (OR (95%CI) = 1.09(1.05 to 1.14), *p* = 2.6 × 10^−5^) (Table 2, Fig. 2, Supplementary Table S2). The association with autism yielded the largest effect size, followed by pervasive developmental disorders (OR (95%CI) = 1.15 (1.10 to 1.20), 2.7 × 10^−7^).

#### Conditioning on BMI

When BMI was added as a covariate in the PheWAS, all previously significant associations remained largely unchanged (Supplementary Table S3).

#### Conditioning on autism diagnosis

When autism was added as a covariate in the PheWAS, all previously significant associations remained largely unchanged, apart from the associations with autism and pervasive developmental disorders which, as expected, were no longer significant (Supplementary Table 4).

#### Sex-stratified PheWAS

**Fig. 3:**
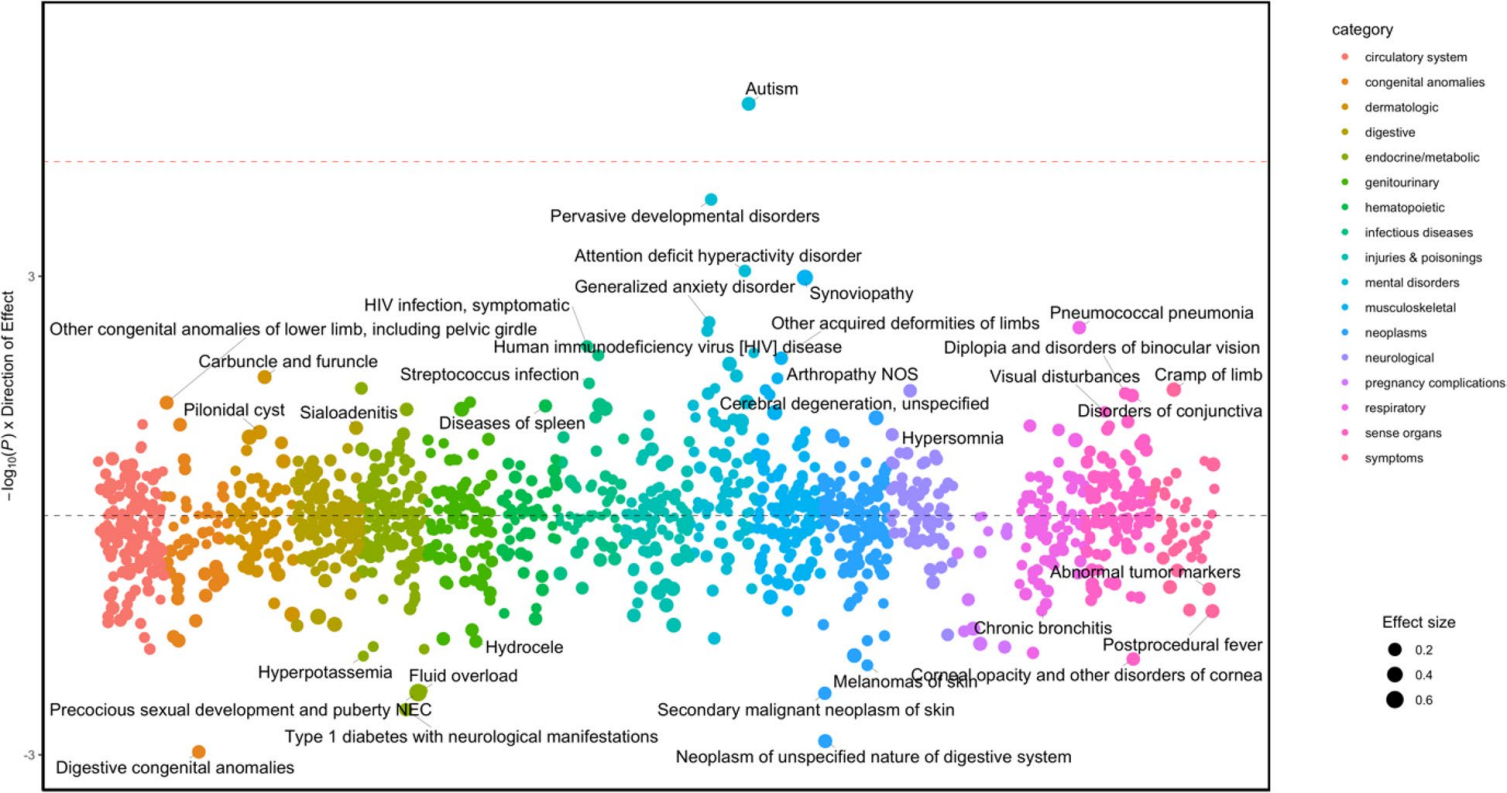
Manhattan plot of autism PGS in males of European genetic ancestries. The y-axis shows the -log10 transformed p-values multiplied by the direction of effect. The dots represent the phecodes which are grouped along the *X* axis by phecode category. Size of the dot corresponds to the effect size. The colors of the dots indicate the phecode categories, which are explained in the figure. The dotted red line signifies the Bonferroni-corrected threshold for statistical significance

**Fig. 4:**
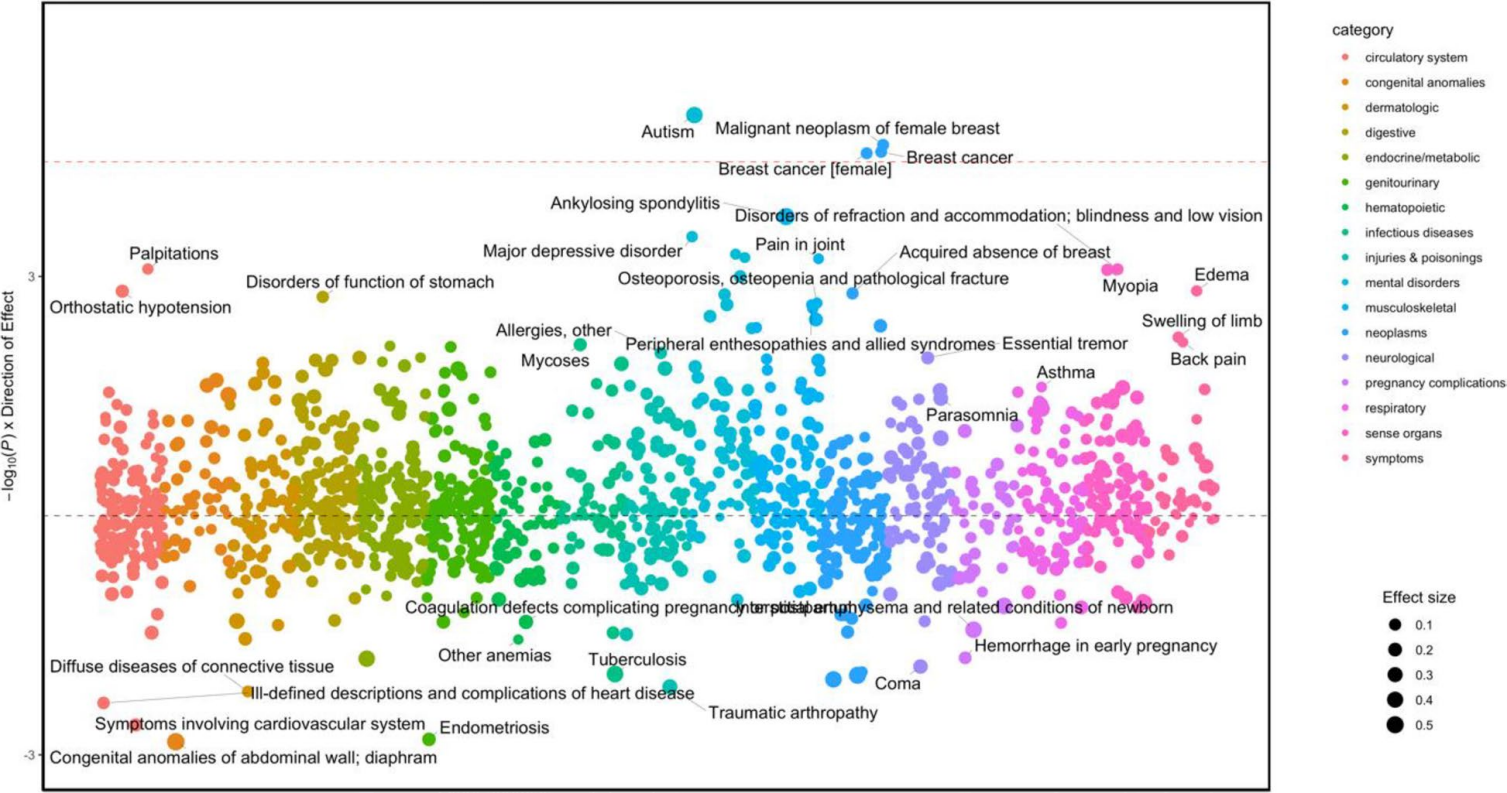
Manhattan plot of autism PGS in females of European genetic ancestries. The y-axis shows the -log10 transformed p-values multiplied by the direction of effect. The dots represent the phecodes which are grouped along the *X*-axis by phecode category. Size of the dot corresponds to the effect size. The colors of the dots indicate the phecode categories, which are explained in the figure. The dotted red line signifies the Bonferroni-corrected threshold for statistical significance

Autism PGS was associated with autism in males (OR (95%CI) = 1.28(1.17 to 1.38), *p* = 6.9 × 10^−6^) (Table 3, Supplementary Table 5, Fig. 3) and females (OR (95%CI) = 1.56(1.36 to 1.75), *p* = 9,4 × 10^−6^) (Table 3, Supplementary Table 6, Fig. 4). In females, autism PGS was also associated with mood disorders (OR (95%CI) = 1.09 (1.06 to 1.12), *p* = 9.3 × 10^−10^), depression (OR (95%CI) = 1.09 (1.06 to 1.12), *p* = 3.8 × 10^−8^), malignant neoplasm of female breast (OR (95%CI) = 1.10 (1.05 to 1.14), *p* = 2.23 × 10^−5^), and breast cancer (OR (95%CI) = 1.09 (1.05 to 1.14), *p* = 2.7 × 10^−5^). The sex-interaction test indicated that there was no statistical evidence of differences between the sexes once the baseline prevalence of the condition in the sample was taken into account (Table 3, Supplementary Table S7). However, we do observe that the ORs for autism PGS are notably higher in females with an autism diagnosis (OR = 1.56) compared to males with an autism diagnosis (OR = 1.28) suggesting that autistic females may have to reach a higher threshold of genetic likelihood before they come to clinical attention.

**Table 3:** Sex-stratified results in the European genetic ancestry population

Sex	Bonferroni significant associations? (yes/no)	Phenotype	OR	95%CI	*P* value
Males	Yes	Autism	1.28	1.17 to 1.38	6.9 × 10^−6^
Females	Yes	Mood disorders	1.09	1.06 to 1.12	9.3 × 10^−10^
		Depression	1.09	1.06 t o 1.12	3.8 × 10^−8^
		Autism	1.56	1.36 to 1.75	9.4 × 10^−6^
		Malignant neoplasm of female breast	1.10	1.05 to 1.14	2.2 × 10^−5^
		Breast cancer	1.09	1.05 to 1.14	2.7 × 10^−5^
		Breast cancer [female]	1.10	1.05 to 1.14	2.8 × 10^−5^
Interaction analyses	No				

*Abbreviations*: *OR* odds ratio, *95%CI* 95% confidence intervals

### Age-stratified PheWAS

#### Birth–11-year age group

Autism PGS was associated with autism diagnosis in this age group (OR (95%CI) = 1.28 (1.16 to 1.40), *p* = 3.0 × 10^−5^) (Table 4, Supplementary Table 8). No other associations surpassed the Bonferroni significance threshold.

**Table 4:** PheWAS results by age-group (European genetic ancestry population)

Age group (years)	Bonferroni significant associations (yes/no)	Phenotype	OR	95%CIs	*p* value
0 to 11	Yes	Autism	1.28	1.16 to 1.40	3.0 × 10^−5^
12 to 18	Yes	Autism	1.36	1.22 to 1.50	1.9 × 10^−5^
19 to 25	No				
26 to 40	Yes	Mood disorders	1.12	1.07 to 1.17	1.1 × 10^−5^
		Depression	1.13	1.07 to 1.19	3.4 × 10^−5^
41 to 60	Yes	Mood disorders	1.09	1.06 to 1.13	7.7 × 10^−8^
		Depression	1.09	1.05 to 1.12	2.8 × 10^−6^
		Electrolyte imbalance	0.93	0.89 to 0.96	2.8 × 10^−5^
61 to 100	No				

*Abbreviations*: *OR* odds ratio, *95%CI* 95% Confidence intervals

#### 12–18-year age group

Similar to the 0 to 11 age group, autism PGS was associated with autism (OR (95%CI) = 1.36 (1.22 to 1.50),* p* = 1.9 × 10^−5^) (Table 4, Supplementary Table S9). No other associations surpassed the Bonferroni significance threshold.

#### 19 to 25-year age group

There was no evidence for associations in this age group (Table 4, Supplementary Table S10).

#### 26 to 40-year age group

Autism PGS was associated with mood disorders (OR (95%CI) = 1.22 (1.07 to 1.17, *p* = 1.1 × 10^−5^), and depression (OR (95%CI) = 1.13 (1.07 to 1.19), *p* = 3.4 × 10^−5^) in this age group (Table 4, Supplementary Table S11).

#### 41 to 60-year age group

Autism PGS was associated with mood disorders (OR (95%CI) = 1.09 (1.06 to 1.13, *p* = 7.7 × 10^−8^) and depression (OR (95%CI) = 1.09 (1.05 to 1.12), *p* = 2.8 × 10^−6^), while there was a negative association with electrolyte imbalance (OR (95%CI) = 0.93, 0.89 to 0.96), *p* = 2.8 × 10^−5^) (Table 4, Supplementary Table S12).

#### 61 to 100-year age group

There was no evidence for associations in this age group (Table 4, Supplementary Table S13).

## Discussion

Our study is the first to test the associations of autism PGS with psychiatric and medical conditions in an EHR context in a tertiary health care setting. There were seven medical diagnoses associated with autism PGS in the EU participants, including autism. These findings suggest that autism, as defined by ICD codes in the EHR, does reflect the genetic architecture of autism as defined in other independent samples. We did not estimate this association in the AF participants, as there were only 59 participants with an ICD diagnosis of autism in this sample (compared to 476 in the EU sample), and the minimum number we set for including case patients in the PheWAS is 100.

Autism PGS was also associated with mood disorders in the EHR, an association that is not surprising given that autism is both phenotypically and genetically correlated with depressive symptoms and major depression [13, 21]. Our study is the first to show that this association is not as strong in childhood and adolescence as it is in adulthood. This could be explained by the fact that the age of onset of mood disorders ranges from mid to late adolescence to the early 40 s, with the median being around the early to mid-20 s [19]. As a result of this, the sample size of mood disorders differs across the different age groups. For instance, the number of cases of mood disorders in adulthood are twice the number of cases in adolescence. Although women are twice as likely to be diagnosed with depression [4], most but not all the genetic background related to depression is shared across sexes [18]. Our results are consistent with this pattern (i.e., the association between the autism PGS and mood disorders is not different across sexes). Our results contrast with a previous autism PGS–PheWAS study in 334,976 participants from the UK biobank that did not find evidence for associations between autism PGS and depression. One limitation that the authors of that study noted is that the UK Biobank participants are less likely to be diagnosed with psychiatric disorders compared to the general UK population, and this may be a potential explanation the authors did not find associations between the autism PGS and any psychiatric-related outcome [22]. Importantly, the links with mood disorders remained significant even after adjusting for an autism diagnosis. While this observation is consistent with pleiotropy it is important to note that the adult diagnostic rate was low (0.7%) and undiagnosed autism may be a confounding factor. Indeed, prior studies find that adults with undiagnosed autism, and thus less access to accommodations and resources, are also more likely to engage in camouflaging [24] and that camouflaging increases risk for mood disorders and anxiety [17].

Autism PGS was associated with a slight increase in breast cancer risk in our study. While epidemiologically there is limited evidence of an association between autism and cancer [8], there is known genetic overlap between these two conditions [7]. For example, the tumor suppressor gene, the Phosphatase and Tensin Homolog (PTEN), has been implicated in both autism [14, 23] and a range of cancers, including breast cancer [31]. Despite the known links between BMI and autism [16] and BMI and breast cancer [27], the associations between autism PGS and breast cancer remained virtually unchanged after adjusting for BMI. Our findings indicate that genes involved in autism may also increase risk for breast cancer later in life. Few studies have focused on the medical conditions of adults with developmental disabilities including autism. Thus, additional research is needed to replicate and extend our findings.

One limitation of our study is the low sample size of the AF participants that likely affected our power. Similarly, sample size difference across age groups may also have reduced power and increased the type II error rate. Another limitation of our study is that our data comes from a single medical center, Vanderbilt University Medical Center, located in Nashville, Tennessee. Thus, the data included in our study is unlikely to be representative of the entire population. However, it should be noted that our study included a large and diverse sample of patients due to the broad catchment of the Vanderbilt Affiliated Health Network (VHAN) which includes outpatient clinics in Tennessee and eight bordering states. As with all research collections, there is a sampling bias. In this case, the sampling bias includes individuals who seek medical care at one or more Vanderbilt affiliated clinics. For example, individuals with reliable transportation, close proximity to VHAN clinics, and resources to cover the cost of medical care are more likely to be represented in the data. Importantly, the VHAN child and adolescent clinics accept both public and private health insurance which reduces the impact of insurance status on entry into the medical system for children. Nevertheless, there are limits to the generalizability of our findings because of the sampling strategy. Collider bias could result from selection of people into a health care setting. However, this is only likely to have a significant impact on the results shown here if autism polygenic scores are also correlated with the sample selection. To the best of our knowledge, our biobank is not enriched for individuals with a family history of autism, mood disorders and/or breast cancer, nor are individuals with these conditions more likely to participate in the biobank. Additionally, autism polygenic scores are not clinically assessed, which reduces the potential for PGS to influence health care, thus reducing the potential for collider bias. Lastly, with regards to ascertainment bias, it is possible that individuals with autism are more likely to be given certain additional diagnoses. For instance, individuals with autism are more likely to be coded with developmental delays and may be more likely to come to clinical attention for other behavioral or psychiatric conditions that are often diagnosed and documented in the EHR. However, it should be noted that we did not specifically restrict our sample to individuals with autism. Instead, we examined the autism polygenic score across the entire biobank, which again reduces the potential for ascertainment bias as the only ascertained variables included entry into the EHR and the biobank. Lastly, we also adjusted for potential confounding factors such as age, sex, and the first 10 principal components of genetic ancestry. Finally, future studies in other cohorts/datasets are needed to replicate our findings.

## Conclusions

Our study is the first to examine the associations between autism PGS and psychiatric and medical conditions in the EHR of a tertiary care center. We replicated the association between autism PGS and autism, and also identified associations with mood disorders and breast cancer. Our findings indicate that genes involved in autism may also increase risk for breast cancer later in life. Future studies are essential to replicate our findings.

## Supplementary Information

**Additional file 1:** **Supplementary Table S1.** PheWAS results of the African American genetic ancestry population. **Supplementary Table S2.** PheWAS results of the European genetic ancestry population. **Supplementary Table S3.** PheWAS results conditional on BMI in the European genetic ancestry population. **Supplementary Table S4.** PheWAS results conditioning on autism diagnosis. **Supplementary Table S5.** PheWAS results in males of European genetic ancestry. **Supplementary Table S6.** PheWAS in females within the European genetic ancestry population. **Supplementary Table S7.** Results of sex-interaction analysis in the European genetic ancestry population. **Supplementary Table S8.** PheWAS results in the 0 to 11 age group (European genetic ancestry population). **Supplementary Table S9.** PheWAS results in the 12 to 18 age group (European genetic ancestry population). **Supplementary Table S10.** PheWAS results in the 19 to 25 age group (European genetic ancestry population). **Supplementary Table S11.** PheWAS results in the 26 to 40 age group (European genetic ancestry population). **Supplementary Table S12.** PheWAS results in the 41 to 60 age group (European genetic ancestry population). **Supplementary Table S13.** PheWAS results in the 61 to 100 age group (European genetic ancestry population).

## Data Availability

Due to data sharing restrictions related to privacy concerns in the EHR, the datasets generated from our hospital population will not be publicly available; however, all scripts used in the study are available upon request.
